# Distance between Anterior Commissure and the First Tracheal Ring: An Important New Clinical Laryngotracheal Measurement 

**Published:** 2015-05

**Authors:** Ehsan Khadivi, Mohammad Ali Zaringhalam, Kamran Khazaeni, Mehdi Bakhshaee

**Affiliations:** 1 *Sinus and Surgical Endoscopic Research Center, Ghaem Hospital, Faculty of Medicine, Mashhad University of Medical Sciences, Mashhad, Iran.*; 2 *Department of Otorhinolaryngology-Head & Neck Surgery, Faculty of Medicine, Mashhad University of Medical Sciences, Mashhad, Iran.*

**Keywords:** Airway stenosis, Anterior commissure, BMI, Subglottic, Trachea, Vocal cord

## Abstract

**Introduction:**

The distance between the anterior commissure of the larynx and the first tracheal ring (AC.T. distance) is of great importance in laryngotracheal surgeries. The amount of narrowing of the subglottic airway is used as a quantitative mean to determine whether the lesion is subglottic or has extended to the trachea and therefore helps in the prediction of the final prognosis.

**Materials and Methods:**

In this study, the larynx was exposed by direct laryngoscopy under general anesthesia. The case was considered to be difficult because the exposure did not optimally reveal the anterior commissure, therefore a cricoid tape or anterior commissure laryngoscope was used. A zero degree Hopkins lens was used to view the anterior commissure and the first tracheal ring. Special markers were used to mark the two points with the distance between those being considered as the AC.T. distance. The relationship between AC.T. distance and the patient's age, sex, BMI, and laryngeal exposure condition during laryngoscopy was also studied.

**Results:**

Eighty-two patients participated in this study. The mean AC.T. distance was measured and was found to be 32.67±3.34 mm in males and 29.80± 3.00 mm in females. This difference was statistically significant between the two groups (P<0.05). There was no statistically significant relationship between BMI, age, laryngeal exposure condition, and the AC.T. distance.

**Conclusion:**

The AC.T. distance was measured to be around 3 cm; with males measuring greater than females. However, future studies may lead to a more accurate practical scale for laryngotracheal surgeries due to possible technical or human errors, in addition to racial differences.

## Introduction

Laryngotracheal surgery has experienced considerable enhancement in recent decades with the development of new surgical techniques and endoscopic procedures, which are replacing the old methods. Laryngotracheal stenosis represents one of the most challenging pathologies confronted in the otolaryngology field. The success rate in the management of airway stenosis has increased with the development of new techniques; leading to a better post-operative decanulation rate (1,2). Therefore, it is critical for diagnostic preoperative endoscopies to determine the length and level of airway pathologies and their extension into adjacent areas.

There are a number of indices reported in literature such as tracheal length, tracheal diameter, and number of tracheal rings with respect to age and sex (3). Distance between the anterior commissure of the true vocal cords and the first tracheal ring (AC.T. distance) is an important fixed clinical landmark during laryngoscopy and is often neglected to be measured and reported in medical literature or texts. The AC.T. distance seems an important criterion in laryngotracheal surgeries, especially in pathologies which lead to the narrowing of the subglottic airway such as fibrosis and granulation tissue formation. The distance between the lesion and the anterior commissure is a unique and helpful mean in determining the exact location of the lesions and is an important factor in the decision making for a surgery. The AC.T. distance differs from the subglottic region, which begins at a distance that is 5-10 mm inferior to the free margin of the true vocal cord and extends to the inferior edge of the cricoid cartilage(4). The AC.T. distance includes the glottis, subglottic, and fibrovascular membrane and the ligament between the cricoid cartilage and the first tracheal ring. Measuring the distance between the lesion and the anterior commissure, when the mean AC.T. distance is measured, is helpful in determining whether the lesion is in the trachea or is spreading to the subglottic region. During laryngoscopy and bronchoscopy, in patients with airway stenosis and subglottic or tracheal tumors, it is impossible to determine the location of the tumor due to the distorted anatomy caused by the pathology in that region. The decanulation rate is less than 90% for laryngotracheal stenosis grade I (stenosis in subglottic and trachea less than 1 cm) and grade II (subglottic stenosis in the cricoids ring over 1 cm) following LTR (laryngotracheal reconstruction), 80% for grade III stenosis (subglottic and upper trachea stenosis), and 50% for grade IV stenosis (subglottic stenosis and glottis involvement) (5). The location of the tumor may change the surgical approach: for tumors located in the trachea resection with end-to-end anastomosis is done; however, for subglottic tumors a total laryngectomy is needed to gain a free margin (6). Therefore, it seems necessary to define the mean AC.T. distance for the normal population to determine the distance between the lesion and its location and utilize that in pre-operative surgical planning.

## Materials and Methods

**Fig 1:**
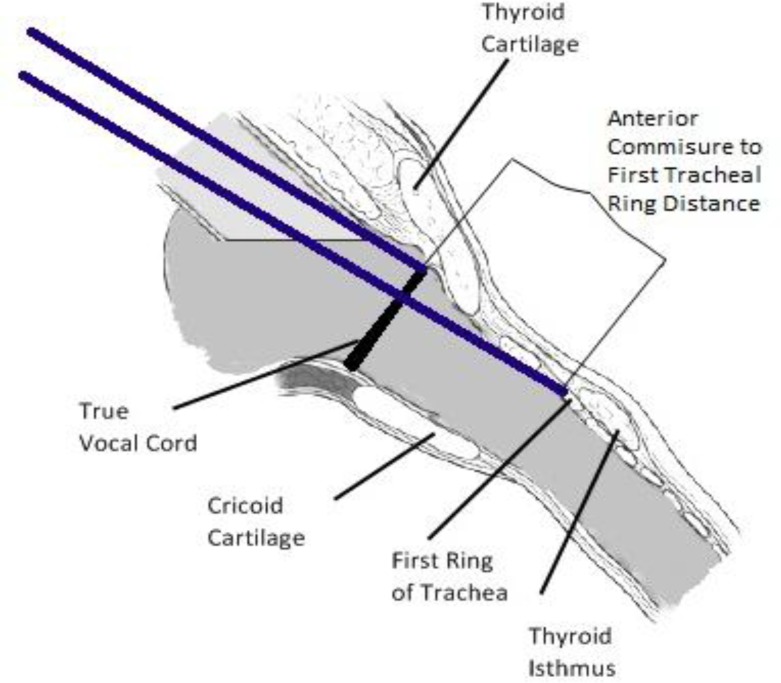
Schematic view of the measurement between anterior commissure and the first tracheal ring.

In this study, the larynx was exposed by direct laryngoscopy under general anesthesia in patients undergoing microlaryngeal surgery for benign TVCS’ lesions. The case was considered to be difficult because the exposure did not optimally reveal the anterior commissure; therefore, a cricoid tape or anterior commissure laryngoscope was used. Both methods were used to expose the larynx in very difficult cases. Then, a 0 degree laryngoscopic lens was used to touch the anterior commissure, which was marked by a marker on the lens. The beginning of the first tracheal ring was then reached and marked with the marker as well. The distance between the two markings was considered to be the AC.T. distance (Fig. 1). In more difficult cases, a Storz triangle anterior commissure laryngoscope was used to investigate the anterior commissure. A caliper was used to measure this distance. 

Informed consent forms approved by the Ethics Committee of Mashhad University of Medical Sciences, along with fingerprints and signature, were taken from the patients.

SPSS version 13 was used for statistical analysis including Pearson Correlation Coefficient, which allows for correlation between two normal quantitative variables. 

In addition, t-test and analysis of variance (ANOVA) were used to compare means of a quantitative variable between two and three groups respectively. Multiple comparisons Duncan test was used as a Post Hoc test. All analyses were two-tailed with significant level (P-value) less than 0.05. Only p-value is presented as a result. Data for demographic variables were summarized using count, percentages, minimum, maximum, mean, and standard deviation. 

## Results

Eighty-two patients with a mean age of 43.11±13.18 (range 23-77), including 25 females and 57 males, were entered in this study. The mean distance between the anterior commissure and the first tracheal ring (AC.T. distance) in these patients was 31.79 ± 3.48, with a minimum distance of 25 mm and a maximum distance of 40 mm. The mean BMI of the subjects was 26.46 ± 5.24, with a lowest BMI of 15.26 and a maximum BMI of 48.24.

The AC.T. distance was significantly related to sex (P<0.05), while other variables including BMI, age, and laryngeal exposure type did not show any statistically significant correlation (Table.1,2).

**Table 1:** Correlation of variables with the distance between anterior commissure and the first tracheal ring in subjects.

**Variables**		**Number**	**AC.T. Distance (mm)**	**P- value**
			Mean ± SD	
Sex	Female	57	29.80 ± 3.00	< 0.000
	Male	25	32.67 ± 3.34	
Laryngeal Exposure	Normal	41	31.52 ± 3.51	0.743
	Difficult	26	32.12 ±3.49	
	Very Difficult	15	31.85 ± 3.42	

**Table 2:** Correlations between AC.T. distance with age and BMI.

**Variables**	**Min**	**Max**	**Mean (SD)**	**P- value**
Age	23.0	77.0	43.11 ± 13.183	0.141
BMI	15.26	48.24	26.46 ± 5.24	0.868

The distribution of the laryngeal exposure conditions and its relationship to the BMI of the patients are listed in (Table. 3). There was no statistically significant relationship between BMI and sex; but in the cases with normal and difficult laryngeal exposure, the male and female mean BMI was 25.92±4.82 and 27.69±6.02 respectively whereas in very difficult laryngeal exposure cases, the mean BMI was 30.08±7.10. This difference was statistically significant (P=0.010). 

**Table 3:** Correlation of variables with BMI

**Variables**		**Number**	**BMI**	**P- Value**
			Mean ± SD	
Sex	Female	57	27.69 ± 6.02	0.205
	Male	25	25.92 ± 4.82	
Laryngeal Exposure	Normal	41	25.45 ± 4.80	0.010
	Difficult	26	25.97 ± 3.76	
	Very Difficult	15	30.08 ± 7.1	
				

## Discussion

The mean AC.T. distance in these patients was 31.79±3.48 with a significant difference between males and females. In the laryngotracheal region, AC.T. distance, and other factors such as the position of the true vocal cord inside the thyroid cartilage, subglottic size, tracheal length, and tracheal diameter, are considered to be important factors evaluated by a surgeon in order to choose the most suitable preoperative laryngotracheal surgical plan. Complete awareness of the exact location of the pathology as well as its extension is the most important factor for a good laryngotracheal surgical plan with predictable prognosis. 

The literature review/web search through Pubmed and Google Scholar did not find any research study measuring the distance between the anterior commissure and the first tracheal ring in the normal population. In one study by Mr. Friedrich et al (1996) in Austria (7), the dimensions of the larynx were measured in 50 cadaver subjects; however, the AC.T. distance was not directly mentioned in the article. 

There was no statistically significant relationship between BMI and AC.T. distance. The results of this case study for laryngeal exposure showed a significant correlation between BMI and very difficult cases in laryngeal exposure. This finding is indirectly related to intubation condition and is in accordance with other studies which show a relationship between higher BMI values and difficult intubation (8-10). This may be due to the diameter of the neck and thyroid cartilage from the mandible and soft tissue around the airway and is not actually due to the high BMI (8,9). 

Although calcification increases with age in the trachea and airway cartilages, which may reduce the airway elasticity, this study showed that no significant correlation is present between age and AC.T. distance. 

The relationship between sex and AC.T. distance was also examined. A difference was anticipated as previous studies have revealed anatomical differences between males and females. A study done by Austrian Fredrich also showed obvious differences in the larynx of males and females (7). In this study, a statistically significant association between sex and AC.T. distance was showed. In a study done by Hiramoto and Isshiki(11,12), the distance between the superior thyroid notch and the anterior commissure measured 8.6 mm in men and 6.5 mm in women. The distance to the inferior thyroid notch in their study measured 9.5 mm and 6.8 mm in men and women respectively. They concluded that the junction of the anterior commissure is determined to be a little (0.5–1 mm) higher than the middle distance between the superior notch and the lower margin. Friedrich7 showed that absolute dimensions differ significantly in men and women but the relative relationships do not differ. The distance between the superior and the inferior thyroid notch is divided with a ratio of 1:1 in both men and women with the fixation of Broyles’ tendon.

Isshikimeasured the distance between the vocal process and the upper margin of the cricoid cartilage in men and women (12), which was reported to be 8.2 mm and 4.6 mm respectively. In this study there is a statistically significant relationship between BMI and the presence of a difficulty during laryngeal exposure. This is in accordance with the anesthesiological studies evaluating the difficulties of intubation among obese patients and those with a high BMI (13-16).

## Conclusion

The following measurement should be considered as a standard reference to plan laryngeal surgeries: 32.67±3.34 mm in males and 29.80±3.00 mm in females. These measurements can also be a preoperative predictor to see whether one could use conservative methods in laryngeal surgical planning instead of performing more aggressive resections. Obviously, technical or human errors, in addition to racial differences, could result in different measurements by future studies with larger sample sizes, which may even lead to a more accurate and practical scale for laryngotracheal surgeries. 
